# Dual-Tracer Positron Emission Tomography/Computed Tomography with [^18^F]FDG and [^18^F]fluorocholine in a Patient with Metastatic Parathyroid Carcinoma

**DOI:** 10.3390/diagnostics14141548

**Published:** 2024-07-17

**Authors:** Cesare Michele Iacovitti, Marco Cuzzocrea, Lauro Gianola, Gaetano Paone, Giorgio Treglia

**Affiliations:** 1 Division of Nuclear Medicine, Imaging Institute of Southern Switzerland, Ente Ospedaliero Cantonale, 6500 Bellinzona, Switzerland; cesaremichele.iacovitti@eoc.ch (C.M.I.); marco.cuzzocrea@eoc.ch (M.C.); gaetano.paone@eoc.ch (G.P.); 2 Service of Endocrinology, Department of Medicine, Ente Ospedaliero Cantonale, 6850 Mendrisio, Switzerland; lauro.gianola@eoc.ch; 3 Faculty of Biomedical Sciences, Università della Svizzera italiana, 6900 Lugano, Switzerland; 4 Faculty of Biology and Medicine, University of Lausanne, 1015 Lausanne, Switzerland

**Keywords:** positron emission tomography (PET), nuclear medicine, hybrid imaging, dual-tracer, choline, FDG, parathyroid carcinoma

## Abstract

Here, we describe the case of a 43-year-old male patient with a metastatic parathyroid carcinoma who underwent dual-tracer whole-body positron emission tomography/computed tomography (PET/CT) with [^18^F]fluorocholine and fluorodeoxyglucose ([^18^F]FDG) for staging. [^18^F]FDG PET/CT detected multiple cervical and mediastinal lymph nodal lesions with increased tracer uptake, whereas [^18^F]fluorocholine PET/CT detected increased tracer uptake on cervical and mediastinal lymph nodal lesions and bone and lung lesions with a better evaluation of metastatic spread. Due to these imaging findings, the patient underwent systemic treatment with chemotherapy. This case demonstrates the added value of dual-tracer PET/CT in this rare metastatic tumor.

**Figure 1:**
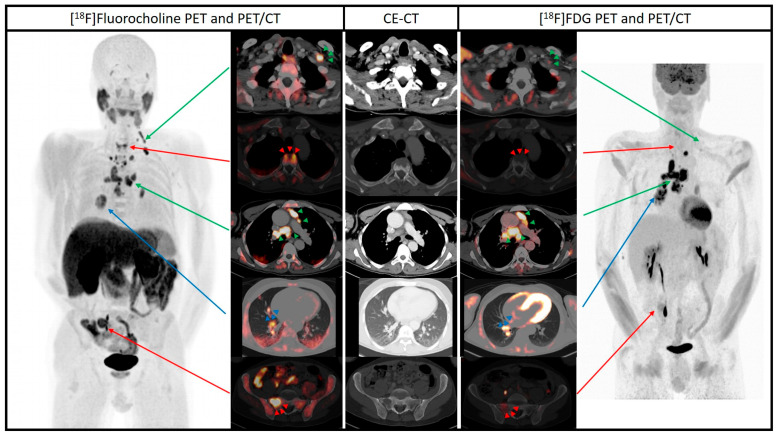
A 43-year-old male patient with a diagnosis of parathyroid carcinoma after left parathyroidectomy underwent dual-tracer PET/CT with [^18^F]FDG and [^18^F]fluorocholine for staging. The delay between the two PET/CT scans was about one month ([^18^F]FDG PET/CT as first examination and [^18^F]fluorocholine PET/CT as second scan). [^18^F]FDG PET/CT was performed 60 min after the injection of 213 MBq of [^18^F]FDG. [^18^F]fluorocholine PET/CT was performed 45 min after the injection of 200 MBq of [^18^F]fluorocholine. CT was performed with the injection of iodinated contrast media. Qualitative criteria for PET image analysis were used for both tracers: areas of increased tracer uptake compared to the background were considered abnormal, excluding the sites of physiological tracer uptake. [^18^F]fluorocholine maximum intensity projection (MIP) PET image and axial fused PET/CT images showed multiple areas of abnormal tracer uptake corresponding to multiple cervical and mediastinal lymph nodal lesions (green arrows), several bone lesions in the spine and the hip (red arrows) and right pulmonary lesions (blue arrows). [^18^F]FDG maximum intensity projection (MIP) PET image and axial fused PET/CT images showed increased tracer uptake in multiple cervical and mediastinal lymph nodal lesions (green arrows). Bone (red arrows) and pulmonary (blue arrows) lesions did not show significant [^18^F]FDG uptake. Due to these dual-tracer PET/CT imaging findings, in particular due to the additional lesions detected by [^18^F]fluorocholine PET/CT compared to [^18^F]FDG PET/CT, a diagnosis of metastatic parathyroid carcinoma with metastatic spread to the lymph nodes, bone and lung was achieved and the patient underwent systemic treatment. Parathyroid carcinoma is a rare endocrine neoplasm with possible metastatic spread. An adequate staging with imaging methods is the mainstay for the choice of treatment [1]. Hybrid imaging methods combine morphological and functional information in the same session. In this regard, [^18^F]FDG PET/CT, the most used PET method in oncology, has been evaluated in patients with parathyroid carcinoma and provides additional information compared to conventional imaging [2]. [^18^F]fluorocholine PET/CT evaluating the membrane cell turnover is an efficient and well-recognized imaging tool to localize parathyroid adenomas [3]. Conversely, scarce information is available on patients with parathyroid carcinoma [4,5,6]. The illustrated case clearly demonstrates a superior advantage of the dual-tracer [^18^F]fluorocholine/[^18^F]FDG PET/CT for the correct evaluation of metastatic spread in patients with parathyroid carcinoma. Even if dual-tracer PET/CT is associated with increased costs and radiation exposure, this imaging approach could be used for staging these rare tumors if both PET radiopharmaceuticals are available. Compared to neuroendocrine and thyroid tumors, there are no prospective studies on dual-tracer PET/CT in parathyroid carcinoma due to the rarity of this disease [7]. Comparative studies on the use of [^18^F]FDG PET/CT and [^18^F]fluorocholine PET/CT are needed to understand which is the best PET tracer for this rare tumor and when dual-tracer PET/CT should be performed.

## Data Availability

The data presented in this article are available on request from the corresponding author.
